# A Reversible Lesion of the Corpus Callosum Associated With Bacterial Meningitis

**DOI:** 10.7759/cureus.25647

**Published:** 2022-06-03

**Authors:** Yasmine Mimouni, Rim Tazi, Amal Miqdadi, Sarra Saaf, Asmaa Hazim

**Affiliations:** 1 Neurology, Cheikh Khalifa Ibn Zayed Hospital, Mohamed VI University of Health Sciences (UM6SS), Casablanca, MAR; 2 Medicine, Cheikh Khalifa Ibn Zayed Hospital, Mohamed VI University of Health Sciences (UM6SS), Casablanca, MAR

**Keywords:** pseudomonas aeruginosa, corpus callosum, spinal anesthesia, meningitis, gram-negative meningitis, obstetrical anesthesia, splenium of the corpus callosum

## Abstract

We report the rare case of a 22-year-old female admitted for headaches, nausea, and an isolated lesion of the splenium of the corpus callosum (SCC) found on brain magnetic resonance imaging (MRI). A lumbar puncture test was performed, and *Pseudomonas aeruginosa* meningitis was found. Twenty days before the symptoms, she had spinal anesthesia for a cesarean section.

As soon as the diagnosis was made, antibiotic therapy was initiated. Nonetheless, no signs or symptoms were related to this lesion, which spontaneously disappeared within one month on the control MRI.

*Pseudomonas aeruginosa* is a gram-negative bacillus and may cause rare and severe meningitis. Therefore, a history of a neurosurgical procedure or spinal anesthesia should be sought in the anamnesis.

## Introduction

Isolated and reversible lesions of the splenium of the corpus callosum (SCC) are usually revealed by brain magnetic resonance imaging (MRI) following mild encephalitis, alcohol toxicity, or even hypoglycemia. However, these radiological lesions were described in some patients with meningitis [1-5].

In our case, bacterial meningitis was diagnosed in a 22-year-old female without any medical issues. The MRI showed an isolated, ovoid, and symmetrical lesion of the SCC, completely reversible after 21 days of dual antibiotherapy, despite a clinical and biological relapse that required a restart of the antibiotherapy with two different antibiotics.

## Case presentation

A 22-year-old female, a mother of three, was admitted to our hospital 15 days after spinal anesthesia was performed for a cesarean section. The patient presented with headaches, a feeling of nausea, and vomiting associated with photophobia and neck pain. The patient also reported bilateral visual blurring and ocular fatigue, without any notion of a decreased visual acuity.

She was afebrile with a good overall condition. The neurological examination found nuchal rigidity with the presence of Kernig's sign and Brudzinski's sign. She was well oriented and presented a good muscle strength and tone, and her deep tendon reflexes were normal. She did not present encephalitis signs whatsoever, and the cranial nerves were no subjects of abnormalities.

The laboratory results were unremarkable except for a low ACE level at 17.4 UI/L and a low vitamin D level at 8.4 ng/mL. The infectious disease workup and immunological investigations (antinuclear antibodies, anti-dsDNA, ANCA, antiphospholipid antibodies, and soluble nuclear antigen-specific antibodies) were negative. However, the lumbar puncture performed on the second day of admission revealed an increased level of proteins at 105 mg/dL and a decreased glucose level at 12 mg/dL. The red blood cell count was at 40, and a pleocytosis at 800 was found, 87% of which were lymphocytes. Gram stain was negative. Given the lymphocytic predominance of the formula and the endemic context of tuberculosis in Morocco, tuberculosis was first suggested as the diagnosis.

In view of this emergency, the patient was put on antibiotics for tuberculosis treatment. A viral origin could not be eliminated at this stage (in particular a herpetic attack); therefore, she received an antiviral medication (acyclovir 640 mg/eight hours). Complementary molecular biology was requested in parallel to the research on meningitis/encephalitis panel and the BK PCR in the cerebrospinal fluid (CSF), both of which came back negative. The culture of cerebrospinal fluid exhibited growth of *Pseudomonas aeruginosa*. There was no intrathecal IgG synthesis found.

**Figure 1:**
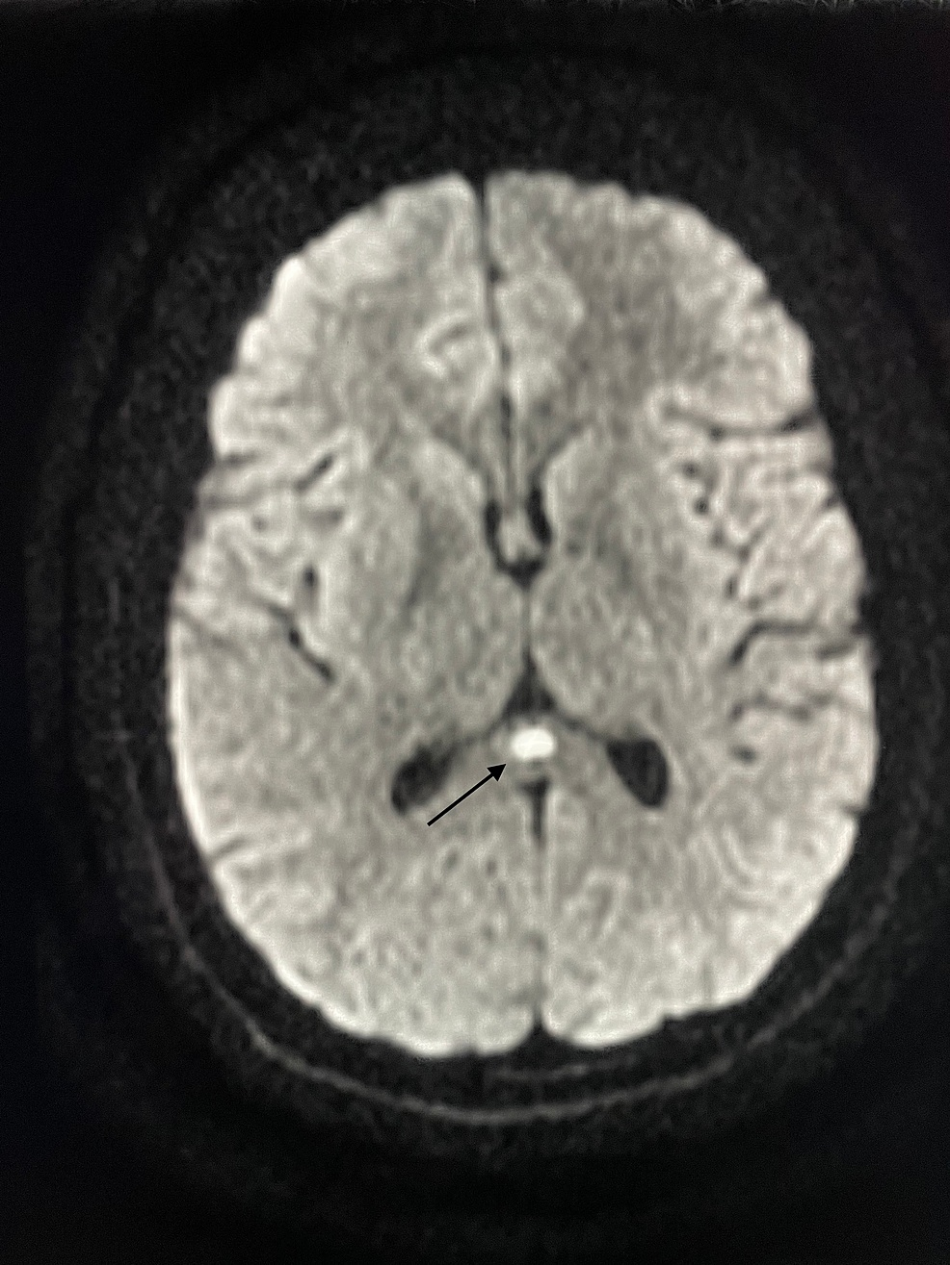
Brain magnetic resonance imaging performed before the antibiotic therapy (axial section, diffusion-weighted imaging (DWI)). DWI shows a focal, isolated, ovoid, hyperintense signal on the splenium of the corpus callosum (arrow).

**Figure 2:**
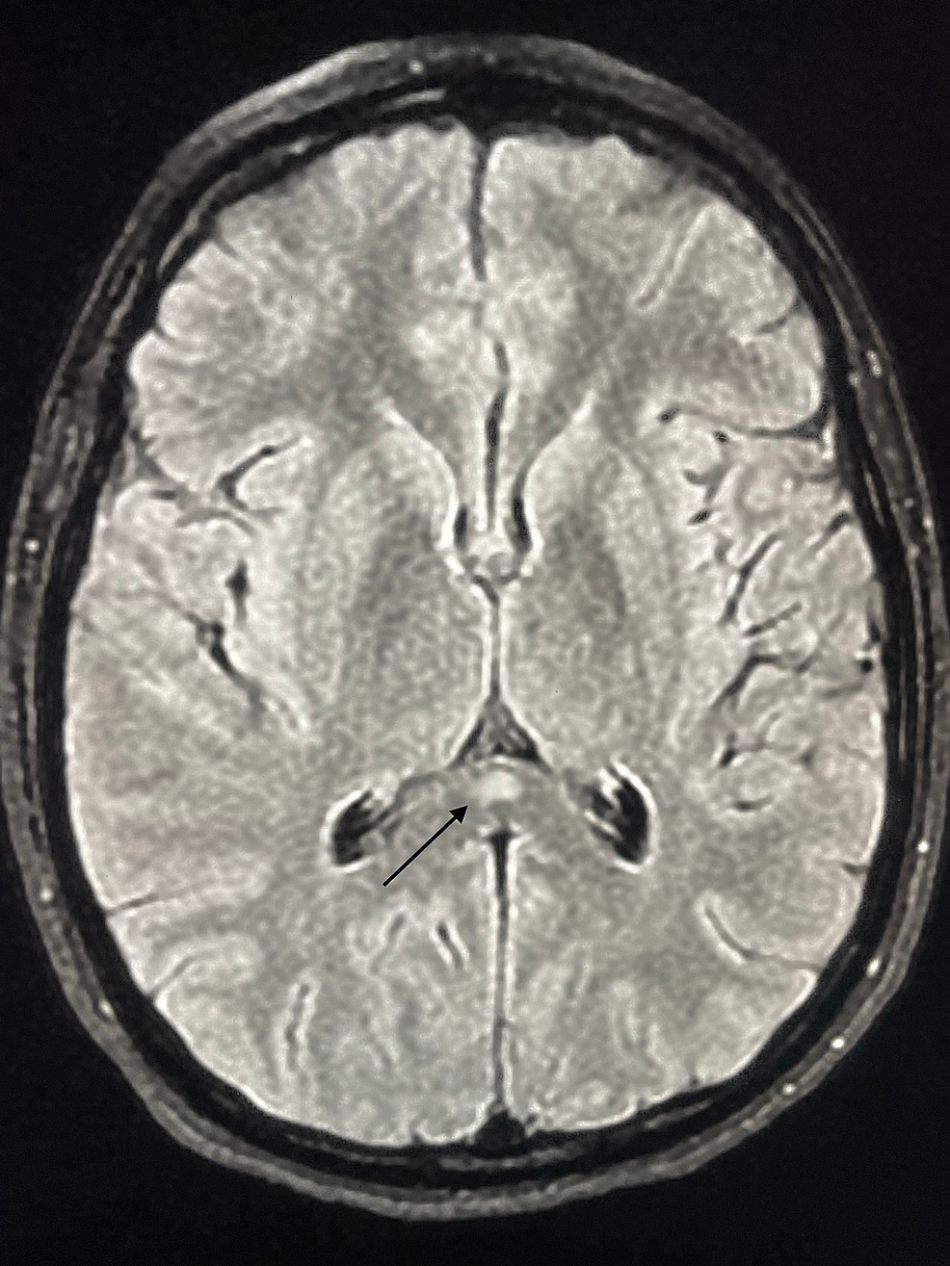
Brain magnetic resonance imaging performed before the antibiotic therapy (axial section, fluid-attenuated inversion recovery (FLAIR) imaging). FLAIR shows a focal, isolated, ovoid, hyperintense signal on the splenium of the corpus callosum (arrow).

**Figure 3:**
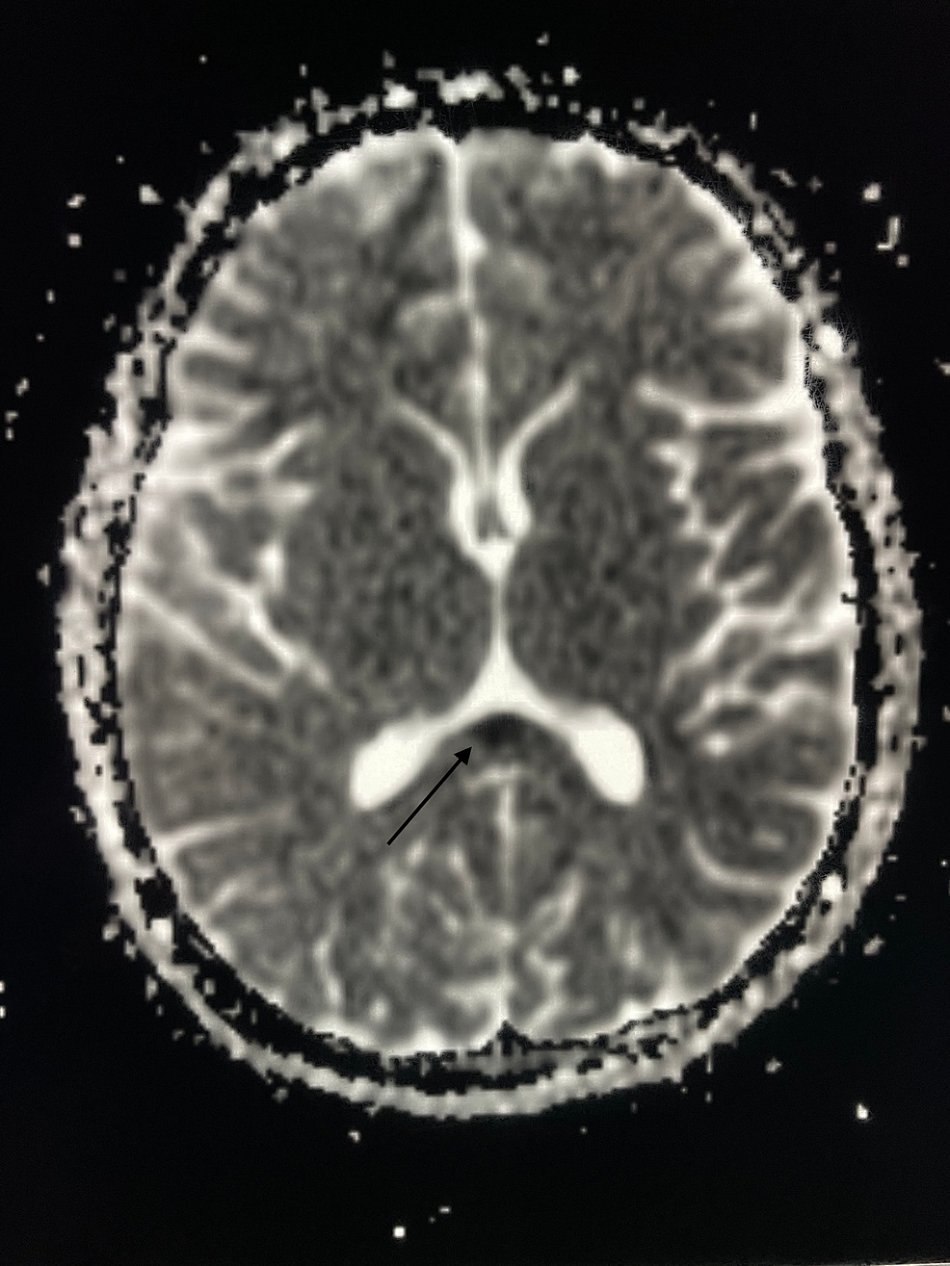
Brain magnetic resonance imaging performed before the antibiotic therapy (axial section, apparent diffusion coefficient (ADC) imaging). ADC shows a focal, isolated, ovoid, hypointense signal (arrow).

**Figure 4:**
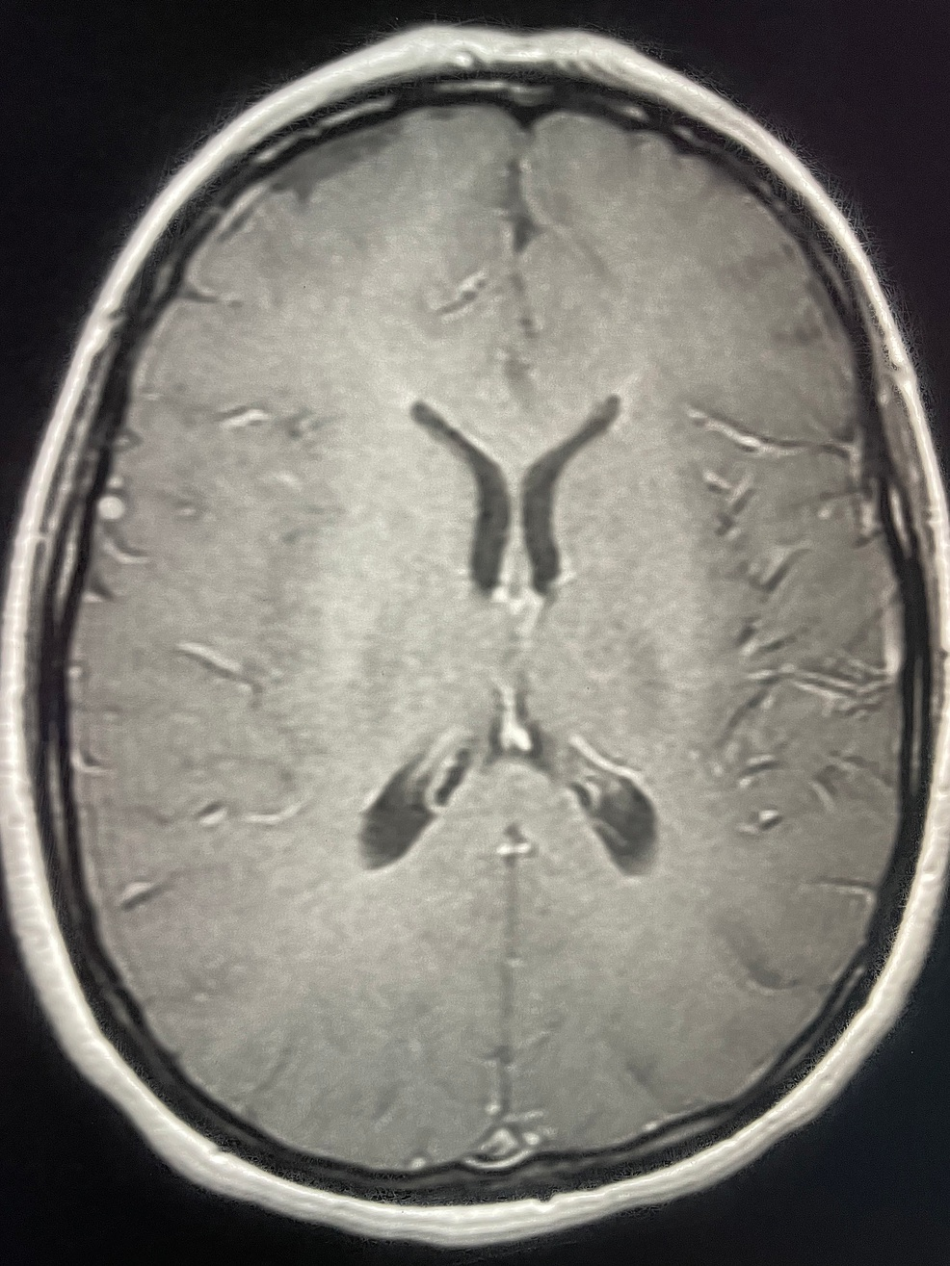
Brain magnetic resonance imaging performed before the antibiotic therapy (axial section, T1-weighted enhanced imaging). The lesion was isointense on the T1-weighted images and was not enhanced by the contrast material.

The brain MRI showed an ovoid lesion of the SCC, characterized by high signal intensity on the diffusion-weighted imaging (DWI) (Figure 1), fluid-attenuated inversion recovery (FLAIR) (Figure 2), and T2 sequences (Figure 3), and low signal intensity on the apparent diffusion coefficient (ADC) map (Figure 4), with no enhancement in gadolinium injected images. No other abnormalities were found.

As soon as the diagnosis was made, the antibacterial and antiviral drugs were stopped, and the patient received a dual antibiotic therapy, made of third-generation cephalosporins (ceftazidime 500 mg ×3/day) and aminoglycosides (amikacin 500 mg ×3/day) for a total period of 21 days.

Good clinical and biological improvement of the patient with neutering cultures on the follow-up lumbar puncture and a progressive decrease in the white blood cell count was seen.

Nonetheless, she became febrile again with intense headache and nausea when the 21-day period was achieved, and the antibiotic was stopped. The culture of the following lumbar puncture performed one day after the clinical relapse showed the presence of *Pseudomonas aeruginosa*.

**Figure 5:**
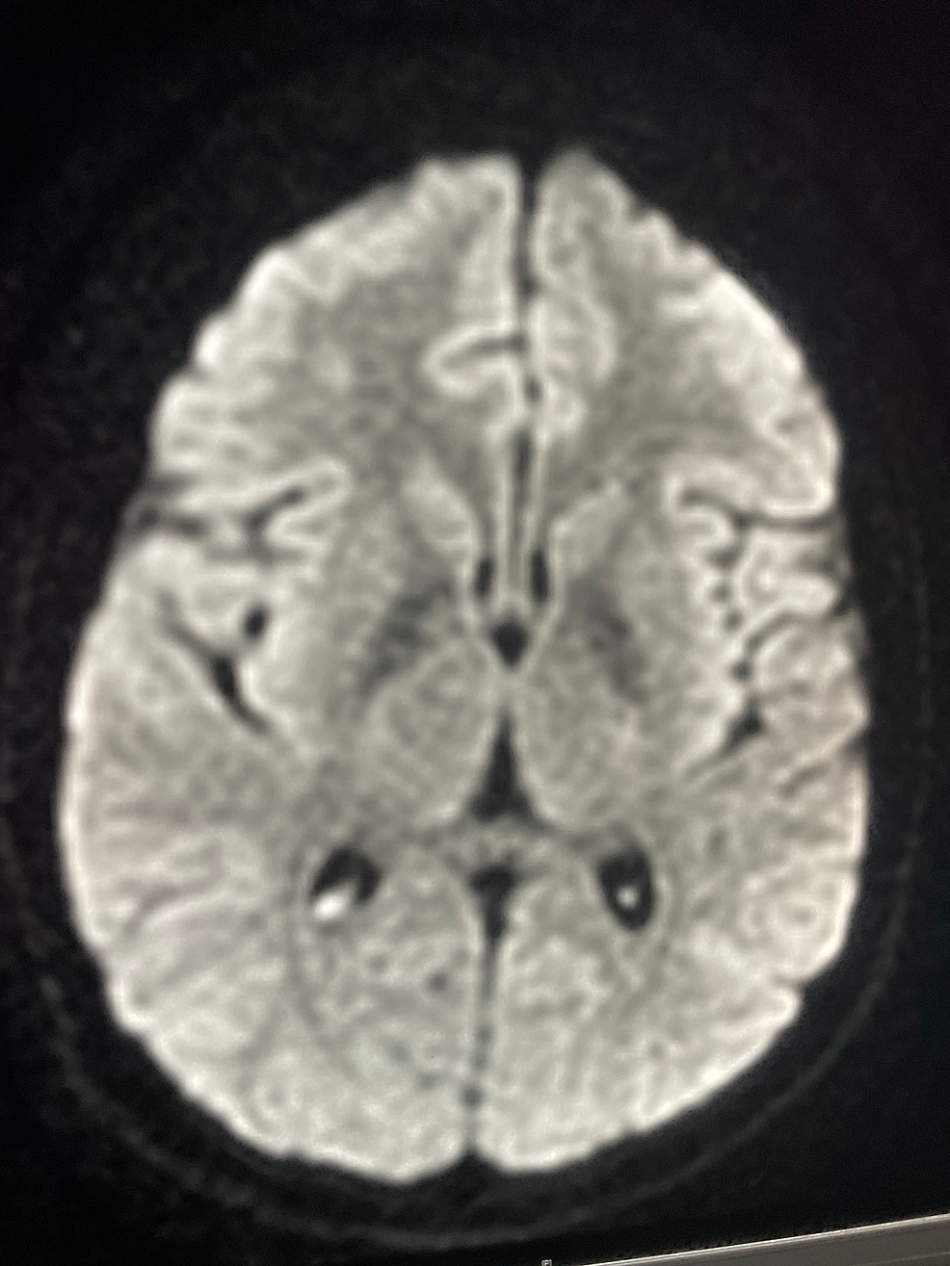
Brain magnetic resonance imaging performed one month after the dual antibiotic therapy (axial section, diffusion-weighted imaging (DWI)). DWI shows the complete disappearance of the splenial lesion.

**Figure 6:**
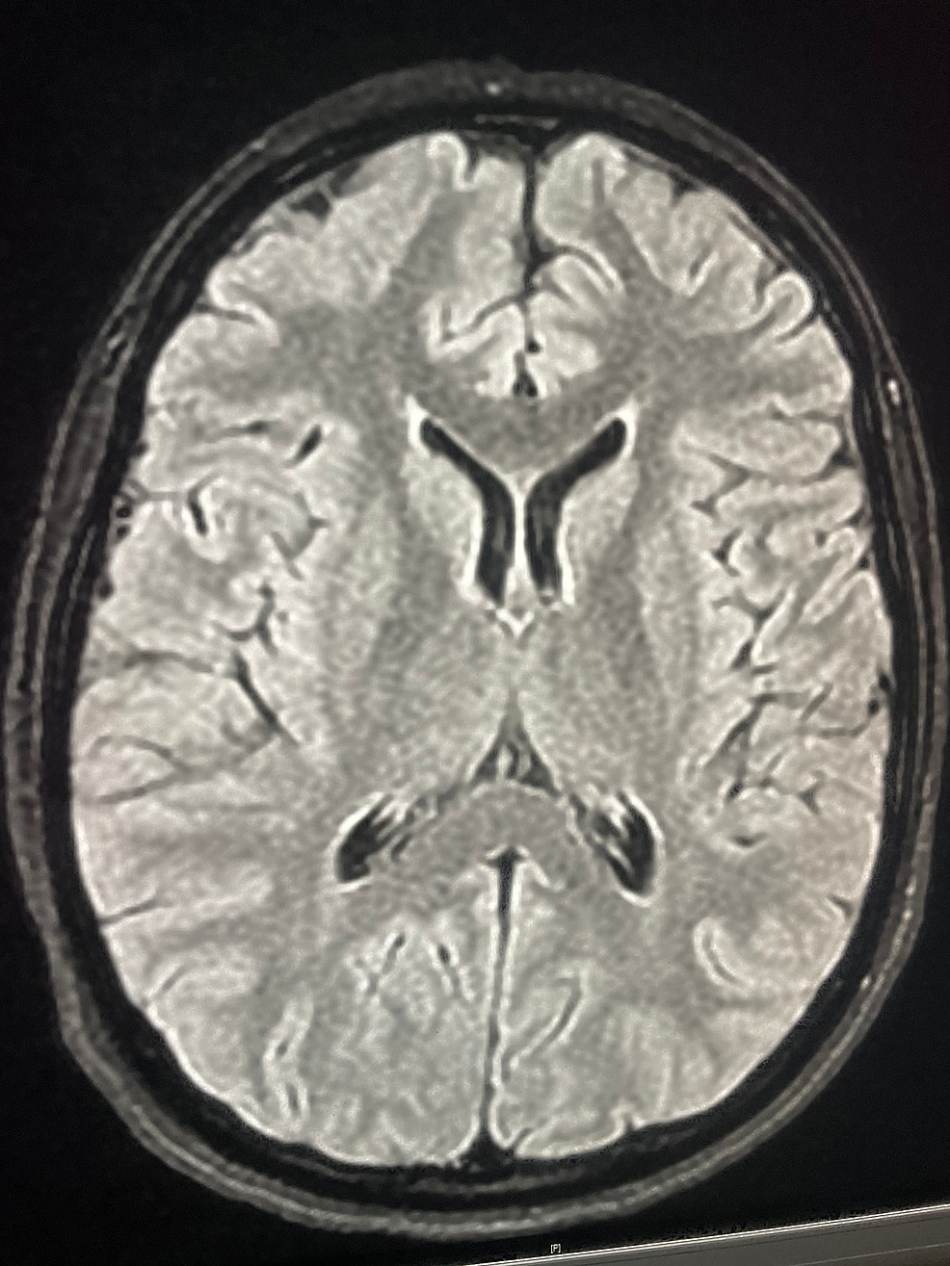
Brain magnetic resonance imaging performed one month after the dual antibiotic therapy (axial section, fluid-attenuated inversion recovery (FLAIR)). FLAIR shows the complete disappearance of the splenial lesion.

However, the repeat MRI performed after the first 21 days of antibiotic was unremarkable with a complete disappearance of the SCC lesion on both the DWI (Figure 5) and FLAIR (Figure 6) sequences, despite the clinical and biological relapse of the patient.

After a multidisciplinary consultation meeting, she was put on another course of dual antibiotic therapy, carbapenem (meropenem 2 g ×3/day) and fluoroquinolones (ciprofloxacin 500 mg twice a day) (Table 1).

Two other CSF analyses were performed, 17 days and 45 days after the start of the carbapenem and fluoroquinolone antibiotics, and showed neutering culture each time with normalization of the white blood cell count (Table 1).

**Table 1:** CSF parameters after withdrawal of the first dual antibiotherapy and clinical relapse.

	Day 1 after clinical relapse	Day 17 after clinical relapse	Day 45 after clinical relapse
Protein level in CSF (mg/dL)	35	32	29
White cell count in CSF (cells/mm^3^)	30	41	<5
PNN level in CSF (%)	5	0	
Lymphocyte level in CSF (%)	95	100	
Culture of CSF	Pseudomonas aeruginosa	Sterile	Sterile
Antibiotic therapy	Meropenem + ciprofloxacin	Meropenem + ciprofloxacin	Ciprofloxacin only

## Discussion

Lesions of the SCC are often diagnosed using magnetic resonance imaging, showing a focal, ovoid, isolated, well-demarcated, and symmetrical lesion, and as high signal intensity in T2, fluid-attenuated inversion recovery (FLAIR), and diffuse-weighted images (DWI) with a decreased signal on the apparent diffusion coefficient (ADC) map [1-3].

Isolated and reversible lesion of the SCC has been reported in cases of encephalopathy (MERS) [4,5], antiepileptic drugs (AED), and/or alcohol toxicity [5], metabolic disorders [4,5] such as hypoglycemia, and reversible posterior leukoencephalopathy syndrome [5].

Cases of isolated reversible splenial lesion in adult meningitis have also been reported due to pathogens including *Streptococcus pneumoniae*, *Legionella pneumophila*, and *Salmonella enteritidis* [2]. However, adults are less affected than children and newborns [5].

The exact cause-and-effect relationship between meningitis and lesions of the corpus callosum has yet to be determined.

Nevertheless, the hyperintensity of the DWI signal may suggest the presence of cytotoxic edema, thus mimicking an ischemic lesion of the corpus callosum, quite rare because of its rich vascularization. Moreover, because of its vascularization, a stroke in this area is often asymmetrical [4]. The reversibility of the lesion is not in favor of cytotoxic edema either.

Therefore, the localization, ovoid symmetrical shape, reversibility, and good prognosis suggest an intramyelinic edema rather than a cytotoxic one [1-5].

In our case, an isolated lesion of the splenium of the corpus callosum was demonstrated before the diagnosis of bacterial meningitis. No other cause that may explain the presence of this lesion was found, in spite of all the complementary examinations conducted.

The normalization of the CSF parameters and the disappearance of the SCC lesion on the follow-up MRI one month after the beginning of the dual antibiotic therapy are in favor of a reversible lesion of the corpus callosum resulting from *Pseudomonas aeruginosa* meningitis.

## Conclusions

An isolated and reversible lesion of the SCC is still a quite rare radiological abnormality, usually found in mild encephalitis/encephalopathy. However, meningitis has to be searched and treated promptly after the diagnosis is made. Although most of the cases are children, adults can be concerned.
